# Functional domains of SP110 that modulate its transcriptional regulatory function and cellular translocation

**DOI:** 10.1186/s12929-018-0434-4

**Published:** 2018-04-11

**Authors:** Jia-Shiun Leu, So-Yi Chang, Chia-Yu Mu, Mei-Ling Chen, Bo-Shiun Yan

**Affiliations:** 1 Institute of Microbiology and Immunology, National Yang-Ming University, Taipei, Taiwan; 2 Institute of Biochemistry and Molecular Biology, National Taiwan University Medical College, Taipei, Taiwan; 3 Graduate Institute of Oncology, National Taiwan University Medical College, Taipei, Taiwan

**Keywords:** Functional domain, Transcriptional regulation, Cellular translocation, Nucleolar localization signal, Nuclear localization signal

## Abstract

**Background:**

SP110, an interferon-induced nuclear protein, belongs to the SP100/SP140 protein family. Very recently, we showed that SP110b, an SP110 isoform, controls host innate immunity to *Mycobacterium tuberculosis* infection by regulating nuclear factor-κB (NF-κB) activity. However, it remains unclear how the structure of SP110 relates to its cellular functions. In this study, we provide experimental data illustrating the protein domains that are responsible for its functions.

**Methods:**

We examined the effects of SP110 isoforms and a series of deletion mutants of SP110 on transcriptional regulation by luciferase reporter assays. We also employed confocal microscopy to determine the cellular distributions of enhanced green fluorescent protein-tagged SP110 isoforms and SP110 mutants. In addition, we performed immunoprecipitation and Western blotting analyses to identify the regions of SP110 that are responsible for protein interactions.

**Results:**

Using reporter assays, we first demonstrated that SP110 isoforms have different regulatory effects on NF-κB-mediated transcription, supporting the notion that SP110 isoforms may have distinct cellular functions. Analysis of deletion mutants of SP110 showed that the interaction of the N-terminal fragment (amino acids 1–276) of SP110 with p50, a subunit of NF-κB, in the cytoplasm plays a crucial role in the down-regulation of the p50-driven tumor necrosis factor-α (TNFα) promoter activity in the nucleus, while the middle and C-terminal regions of SP110 localize it to various cellular compartments. Surprisingly, a nucleolar localization signal (NoLS) that contains one monopartite nuclear localization signal (NLS) and one bipartite NLS was identified in the middle region of SP110. The identification of a cryptic NoLS in the SP110 suggests that although this protein forms nuclear speckles in the nucleoplasm, it may be directed into the nucleolus to carry out distinct functions under certain cellular conditions.

**Conclusions:**

The findings from this study elucidating the multidomain structure of the SP110 not only identify functional domains of SP110 that are required for transcriptional regulation, cellular translocation, and protein interactions but also implicate that SP110 has additional functions through its unexpected activity in the nucleolus.

**Electronic supplementary material:**

The online version of this article (10.1186/s12929-018-0434-4) contains supplementary material, which is available to authorized users.

## Background

Tuberculosis (TB), caused by *Mycobacterium tuberculosis* (*Mtb*) infection, is one of the top ten causes of death worldwide [1]. Approximately one-third of people have been infected with *Mtb*, but only 10% of these individuals develop the clinical disease [2]. Genetic polymorphisms among hosts have been shown to contribute to the outcome of *Mtb* infection in both humans and experimental animal models [3–7]. We have previously identified the *Ipr1* (intracellular pathogen resistance 1) gene, which is located within the *sst1* (supersusceptibility to tuberculosis 1) locus on chromosome 1 (49–54 cM) in mice, as a genetic determinant conferring host innate immunity to *Mtb* infection [8]. *Ipr1* is not expressed in the *sst1* susceptible macrophages that are naturally *Ipr1*-deficient, but is expressed in the *sst1* resistant macrophages in which *Ipr1* expression is up-regulated upon *Mtb* infection. Expression of the *Ipr1* transgene in the *sst1* susceptible macrophages restricted *Mtb* and *Listeria monocytogenes* growth in vitro. These findings indicate that the *Ipr1* gene may function to integrate mechanisms controlling macrophage activation, cell death, innate immunity, and pathogenesis during infection with intracellular pathogens [8, 9].

The gene orthologous to *Ipr1* in humans is *SP110*, which is located on chromosome 2q37.1. Like *Ipr1*, *SP110* expression is intensively regulated by interferons (IFNs), suggesting that the function of *SP110* is related to the immune response [10]. A number of genetic variants of *SP110* have been reported to be associated with susceptibility to human tuberculosis, although the results of studies regarding the relationship between *SP110* polymorphisms and TB susceptibility are inconsistent [11–18]. In addition, genetic mutations in the *SP110* gene have been documented to be responsible for immunodeficiency and familial hepatic veno-occlusive disease [19–21]. SP110 proteins have at least three isoforms, including the more dominantly expressed SP110a, b, and, c isoforms**,** which are believed to be the result of alternative mRNA splicing. It has been demonstrated that SP110b protein physically interacts with viral proteins, such as the hepatitis C virus core protein [22] and the Epstein–Barr virus SM protein [23]. However, the role of SP110 in host immunity to these diseases remains to be further determined.

Protein structural analyses demonstrated that both Ipr1 and SP110 contain an SP100-like nuclear body protein interaction domain (SP100 domain), a chromatin-associating SAND domain (named after SP100, AIRE-1, NucP41/75, DEAF-1), and an LXXLL (where L is leucine and X is any amino acid) nuclear receptor binding motif. The SP100 domain, a region of approximately 100 amino acids, is rich in hydrophobic amino acid residues and may function as a protein-protein interaction domain. The SAND domain, around 80 conserved amino acid residues in length, may function in chromatin-mediated transcriptional regulation [24]. In addition, the SAND domain also possesses a modular structure that allows it to associate with several other modules, such as SP100 domain, bromodomain (BRD), PHD (plant homeodomain)-type and MYND (myeloid, Nervy, and DEAF-1)-type Zn finger domains, which have been shown to be involved in interactions with chromatin or transcription factors. SAND domain-containing proteins have been demonstrated to bind DNA sequences specifically via their SAND domain, and the DNA-binding ability requires a conserved KDWK motif in the SAND domain [24]. The LXXLL-type nuclear receptor binding motif, which has been identified in nuclear receptor co-activators, co-repressors, other transcription regulators, and chromatin proteins, is involved in protein-protein interactions that may accordingly regulate the process of gene transcription and translation [25–27]. The fact that the Ipr1 and SP110 proteins contain these functional domains that are similar to nuclear proteins involved in transcriptional regulation suggests that both proteins may function as transcriptional co-activators/co-repressors and nuclear hormone receptors [28].

In humans, SP110 belongs to the SP100/SP140 family, which includes SP100, SP140, and AIRE-1 proteins [29]. All family members contain the SP100 domain and the SAND domain, and most of the family members also contain chromatin-associating domains, such as PHD, BRD, and HMG (high mobility group) domains. These proteins are named as speckled proteins (SP) because they are not distributed uniformly but form aggregates that are co-localized with a macromolecular structure called nuclear bodies (NBs) in the nucleus [28]. NBs contain SP100/SP140 family and PML (promyelocytic leukemia) proteins, and many NB proteins have been revealed to play a key role in the regulation of transcription, cell division, apoptosis, senescence, and response to DNA damage or infection [30, 31]. Among SP110 isoforms, SP110b is the nearest structural homologue of the Ipr1 protein (41% identity). Our recent study demonstrated that SP110b modulates nuclear factor-κB (NF-κB) activity resulting in the down-regulation of tumor necrosis factor-α (TNFα) production and concomitant up-regulation of NF-κB-induced anti-apoptotic gene expression, thereby suppressing IFNγ-mediated monocyte/macrophage cell death [16]. The present study further dissected the domains of SP110 and defined their functions. The results of our work not only provide important information revealing the biochemical properties of the protein but also facilitate the investigation of currently unknown roles of this protein in cells.

## Methods

### Cell lines and culture

The HEK293T human embryonic kidney cell line and the H1299 human non-small cell lung cancer cell line (ATCC) were cultured in RPMI 1640 medium containing penicillin/streptomycin, 2 mM L-glutamine, and 7.5% tetracycline-free FBS (Life Technologies) at 37 °C in 5% CO_2_. All cell lines were regularly tested for mycoplasma contamination.

### Plasmids

For inducible gene expression, the pHAGE backbone lentiviral vector with the TRE promoter (pHAGE-TRE-eGFP-Ubc-LNGFR) was utilized as previously described [16]. For transient gene expression, two pcDNA3.1 (Life Technologies)-derived vectors, pcDNA-3 × FLAG and pcDNA-3 × HA, were provided as kind gifts from Dr. S.-L. Yu. cDNAs of the full-length human SP110a, SP110b, SP110c, p50 and SP110 deletion mutants were amplified by polymerase chain reaction (PCR) and inserted into the abovementioned vectors. For the TNFα promoter activity assay, an approximately 1.9 kb segment of the TNFα 5′-flanking region (− 1786 to + 175), which encompasses the transcriptional start site, was amplified by PCR and inserted into the pGL3-Basic vector (firefly luciferase; Promega) to generate the pGL3-TNFα promoter-F.Luc plasmid (firefly luciferase). The pSV40-R.Luc plasmid (*Renilla* luciferase; Promega) was used as an internal normalization control for transfection efficiency. The PCR primers that were designed and used for plasmid construction are listed in Additional file 1: Table S1. The PCR primers that were designed and used for generating deletion mutants and site-directed mutagenesis of SP110 are listed in Additional file 1: Tables S2 and S3, respectively.

### Confocal microscopy

Cells were seeded on glass coverslips and transfected with each expression plasmid. To induce protein expression, the cells were treated with 1 μg ml^− 1^ doxycycline (Dox) (Clontech). Two days after transfection, the cells were washed three times with PBS and then fixed with 4% paraformaldehyde in PBS for 10 min, washed three times with PBS, and stained with Hoechst (Sigma) for 10 min. After being washed three times with PBS, the cells were ready for observation. For cells stained with antibodies, cells were permeabilized with ice-cold 0.2% Triton X-100 for 5 min after being fixed with 4% paraformaldehyde in PBS as aforementioned. The cells were then blocked with 0.5% BSA in PBS for 30 min, followed by incubating with primary antibody in blocking solution overnight at 4 °C. The cells were then washed three times with PBS and incubated with the secondary antibodies for 1 h and Hoechst for 10 min. After being washed three times with PBS, the cells were mounted and ready for observation. The cellular distribution of enhanced green fluorescent protein (eGFP)-SP110 fusion proteins and eGFP in cells was observed using a Leica TCS SP5 laser confocal microscope. Besides, the cellular distribution of key eGFP-SP110 fusion proteins was confirmed by expressing FLAG-tagged SP110 proteins in HEK293T cells and subsequent immunofluorescence staining (anti-FLAG primary antibody and Alexa Fluor 488 conjugated secondary antibody) and confocal microscopy (Additional file 2: Figure S1).

### Western blot analysis and immunoprecipitation

Whole-cell lysates and nuclear extracts were prepared according to previously described procedures [16]. Briefly, whole-cell lysates were prepared in RIPA buffer (10 mM sodium phosphate [pH 7.2], 150 mM NaCl, 1% NP-40, 0.25% sodium deoxycholate, and 0.1% SDS). To prepare the nuclear extracts, the cells were lysed in hypotonic buffer (10 mM HEPES [pH 7.9], 10 mM KCl, 1.5 mM MgCl_2_, and 0.2% Nonidet P-40). After centrifugation at 15,000×*g* for 3 min, the nuclei were washed with hypotonic buffer and then extracted with nuclear extraction buffer (20 mM HEPES [pH 7.9], 420 mM NaCl, 1.5 mM MgCl_2_, and 25% glycerol) using a rotary mixer at 4 °C for 2 h. For immunoprecipitation (IP), whole-cell lysates were incubated with anti-FLAG M2 magnetic beads (Sigma-Aldrich) overnight at 4 °C. After washing the beads with PBS, the proteins were eluted by boiling the beads with 1 × sample buffer. All of the buffers were supplemented with 1 × complete protease inhibitor cocktail (EDTA-free) with or without phosphatase inhibitor cocktails (both from Roche Diagnostics). The protein concentrations were measured using a BCA protein assay kit (Pierce Biotechnology). The proteins were separated by 8–12% SDS-PAGE and then transferred to PVDF membranes (Pall) for immunoblotting. All of the membranes were developed using Immobilon Western HRP Substrate (Millipore). The primary antibodies utilized for the immunoblotting are listed in Additional file 1: Table S4.

### Luciferase assays

HEK293T cells were seeded at 4×10^4^ per well (24-well plate) 24 h before transfection, followed by being co-transfected with pGL3-TNFα promoter-F.Luc (200 ng), pSV40-R.Luc (20 ng) and the indicated constructs (200 ng for each) using Lipofectamine 2000 transfection reagent (Life Technologies) and incubated for 24 h. At 24 h post-transfection, medium were replaced with fresh medium, and the cells were cultured in the presence or absence of 25 μM GSK-3β inhibitor VIII (Santa Cruz Biotechnology) for an additional 24 h. Luciferase activities were then determined using the Dual-Glo^®^ Luciferase Assay System (Promega) according to the manufacturer’s instructions, and the luminescent signals were measured using a Victor3 multi-label counter (PerkinElmer). The reporter activity was calculated as a normalized firefly luminescence/*Renilla* luminescence ratio, and the data were presented as the mean ± SD of 3–4 replicates.

### Statistical analyses

Quantitative data were analyzed by two-tailed unpaired *t* test using GraphPad Prism software. *p* values less than 0.05 were considered statistically significant and the number of asterisks represents the degree of significance with regard to the *p* values.

## Results

### The N-terminal region of SP110 plays a major role in the down-regulation of TNFα promoter activity

**Fig. 1:**
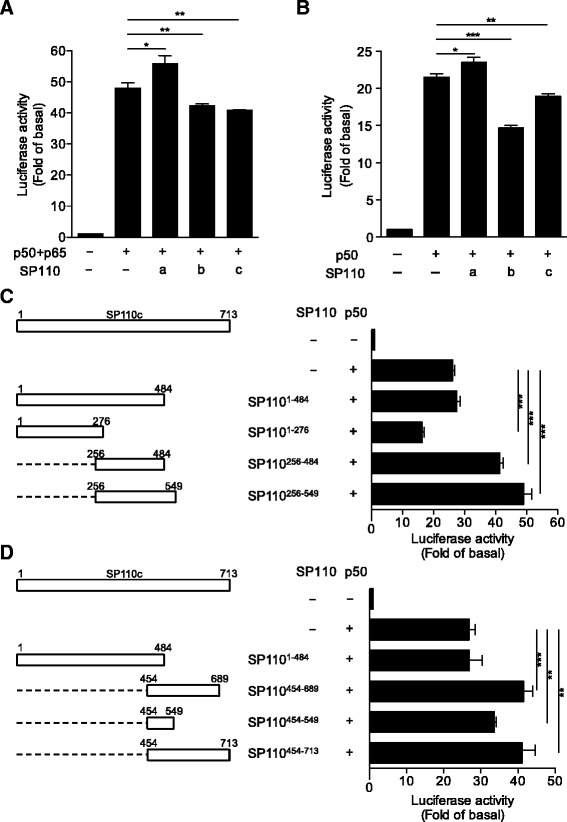
SP110 regulates NF-κB-driven transcription. **a** HEK293T cells were co-transfected with pGL3-p5 × κB-F.Luc, pSV40-R.Luc, and the indicated constructs, and the relative firefly (F.)/*Renilla* (R.) luciferase (Luc) values for a basic promoter with 5 × κB sites were measured 2 days after co-transfection. The empty vector negative control is shown on the left. **b** HEK293T cells were co-transfected with pGL3-TNFα promoter-F.Luc, pSV40-R.Luc, and the indicated constructs, and the relative Luc values for the TNFα promoter were measured 2 days after co-transfection. The empty vector negative control is shown on the left. **c** and **d** HEK293T cells were co-transfected with pGL3-TNFα promoter-F.Luc, pSV40-R.Luc, and the indicated constructs, and the relative Luc values for the TNFα promoter were measured 2 days after co-transfection. The empty vector negative control is shown on the top. In (**a** and **b**), a, b, and c correspond to SP110a, SP110b, and SP110c, respectively. In (**c** and **d**), schematic representation of SP110 deletion mutants is shown on the left. The data are presented as the mean ± SD. Statistical significance of the difference between two sample groups was calculated using a two-tailed unpaired *t*-test. *P < 0.05; **P < 0.01; ***P < 0.001. The experiments were done at least twice

Protein structural analyses demonstrated that SP110 may function as a transcriptional co-regulator [28]. Very recently, we have shown that SP110b interacts with NF-κB and regulates its transcriptional activity [16]. To determine whether SP110 isoforms have different effects on transcriptional regulation, we used a reporter assay system in which the luciferase reporter gene is driven by a basic promoter element (TATA box) linked to 5 NF-κB binding element repeats (κB sites). We demonstrated that SP110b and SP110c significantly decreased promoter activity driven by NF-κB (p50/p65) while SP110a increased this activity (Fig. 1a). Our previous study showed that over-expression of the NF-κB p50 dimer induced the highest levels of TNFα promoter activity [16]. To examine the effects of each SP110 isoforms on p50 dimer-driven TNFα promoter activity, we performed the luciferase reporter assays with the TNFα promoter region and demonstrated that the p50-driven TNFα transcription was suppressed by SP110b and SP110c but slightly enhanced by SP110a (Fig. 1b). The results indicate that these three SP110 isoforms may have distinct cellular functions.

To define the region of SP110 that is critical for SP110-mediated down-regulation of p50 dimer-driven TNFα promoter activity, serial deletion mutants of SP110 were generated, and their effects on TNFα transcription were examined. We found that the N-terminal fragment (amino acids 1–276) of SP110 (1–276, SP110^1–276^) had a similar effect on the down-regulation of TNFα transcription to that of full-length SP110b. However, the middle (256–484, SP110^256–484^; 256–549, SP110^256–549^) and C-terminal (454–689, SP110^454–689^; 454–549, SP110^454–549^; 454–713, SP110^454–713^) regions of SP110 enhanced p50-driven TNFα reporter activity; this was even evident with the C-terminal tail (SP110^454–549^) of SP110b, the SP110 isoform that down-regulates TNFα transcription (Fig. 1c). Together, these results indicate that the N-terminal region of SP110 between amino acids 1–276 plays a key role in the down-regulation of the TNFα promoter activity and the various C-terminal regions of the SP110 isoforms may alter the function of the N-terminal region, suggesting that these C-terminal regions may contribute to the diversity in the functions of distinct SP110 isoforms.

### The middle and C-terminal regions of SP110 influence protein localization

**Fig. 2:**
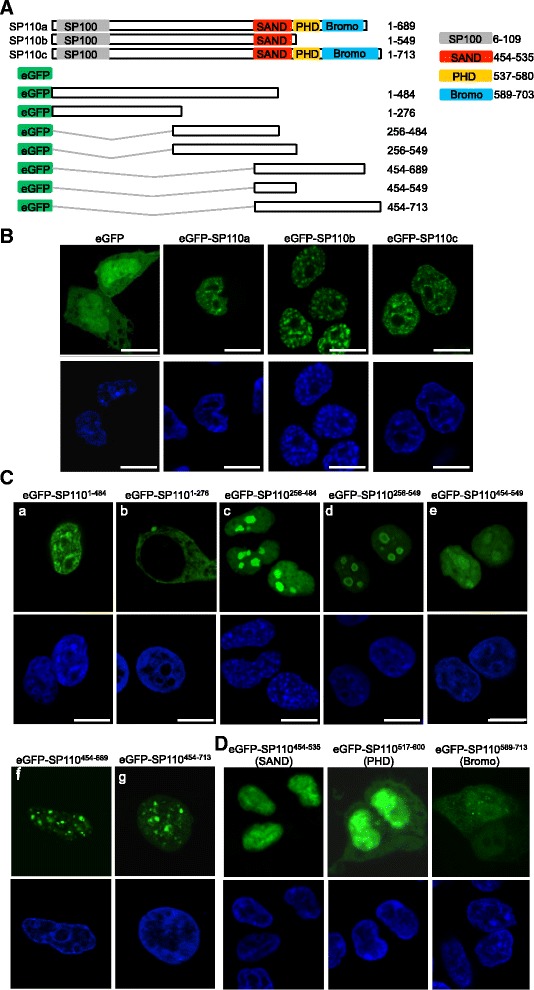
Subcellular localization of eGFP fusion proteins containing wild-type SP110 or SP110 mutants. **a** Schematic representation of eGFP fusion proteins containing SP110 deletion mutants. SP100: SP100 domain; SAND: SAND domain; PHD: PHD finger; Bromo: Bromodomain. **b–d** HEK293T cells were transfected with the indicated constructs, and the cellular distribution of eGFP and eGFP-SP110 fusion proteins (wild-type and mutated forms) was visualized using confocal microscopy at 2 days post-transfection (upper panels). The cells were also subjected to Hoechst staining to identify nuclei (lower panels). Scale bars: 10 μm. The experiments were done at least twice

To explore the roles of the C-terminal regions of the SP110 isoforms, the three isoforms, as well as a series of deletion mutants of SP110, were fused to the C-terminal end of eGFP and expressed in HEK293T cells (Fig. 2a). We first found that the three full-length isoforms of SP110 were localized to the nucleus and formed nuclear speckles as previously documented [28], while eGFP alone was present both in the cytoplasm and the nucleus (Fig. 2b). Nuclear speckles are distinct nuclear sub-compartments and are different from PML-nuclear bodies. SP110 can form nuclear speckles and also localize to PML-nuclear bodies as a PML-nuclear body protein [28]. Furthermore, we observed that the region between amino acids 1–484 of SP110 (SP110^1–484^) had a distribution in the nucleus similar to that of full-length SP110 proteins, but the protein fragment that contains amino acids 1–276 (SP110^1–276^) was localized in the cytoplasm (Fig. 2c, (a) and (b)). This result suggested that nuclear localization signals (NLSs) are present between amino acids 276 and 484 of SP110. To verify this finding, the cellular localization of the middle fragment of SP110 (256–484, SP110^256–484^) was evaluated; however, the results showed that this region was localized to both the nucleolus and the nucleoplasm (Fig. 2c, (c)). In addition, the C-terminal portions of the SP110 isoforms were examined, and the results demonstrated that the fragment of SP110b containing amino acids 256–549 (SP110^256–549^) exhibited the same nucleolar localization as SP110^256–484^, while the C-terminal tail of SP110b (454–549, SP110^454–549^) was mainly localized to the nucleus (Fig. 2c, (d) and (e)). The C-terminal regions of both SP110a and SP110c were also localized to the nucleus and formed larger nuclear speckles than the respective full-length proteins (Fig. 2c, (f) and (g)).

Structurally, the three SP110 isoforms differ only at their C-termini due to the presence of a PHD finger and a BRD module in SP110a and SP110c but not in SP110b, although the BRD module is truncated in SP110a. To investigate the roles of these C-terminal domains in cellular localization, constructs encoding eGFP fused with the SAND domain (amino acids 454–535), PHD finger (amino acids 537–580), or BRD module (amino acids 589–703) were generated from the SP110c cDNA and expressed. We observed that eGFP-SP110^454–535^, which contains the SAND domain, was localized to the nucleus, while eGFP-SP110^517–600^, which contains the PHD finger, was distributed throughout the whole cell (Fig. 2d). Furthermore, eGFP-SP110^589–713^, which includes the BRD module, formed sparse and smaller nuclear speckles in the nucleus, although it was localized throughout the whole cell (Fig. 2d). Taken together, these results indicate that the major nuclear and nucleolar localization signals of SP110 are located within amino acids 256–484 and suggest that the SAND domain and the BRD module might contribute to nuclear targeting and nuclear speckle formation, respectively.

### The nuclear and nucleolar localization signals of SP110 are located in its middle region

**Fig. 3:**
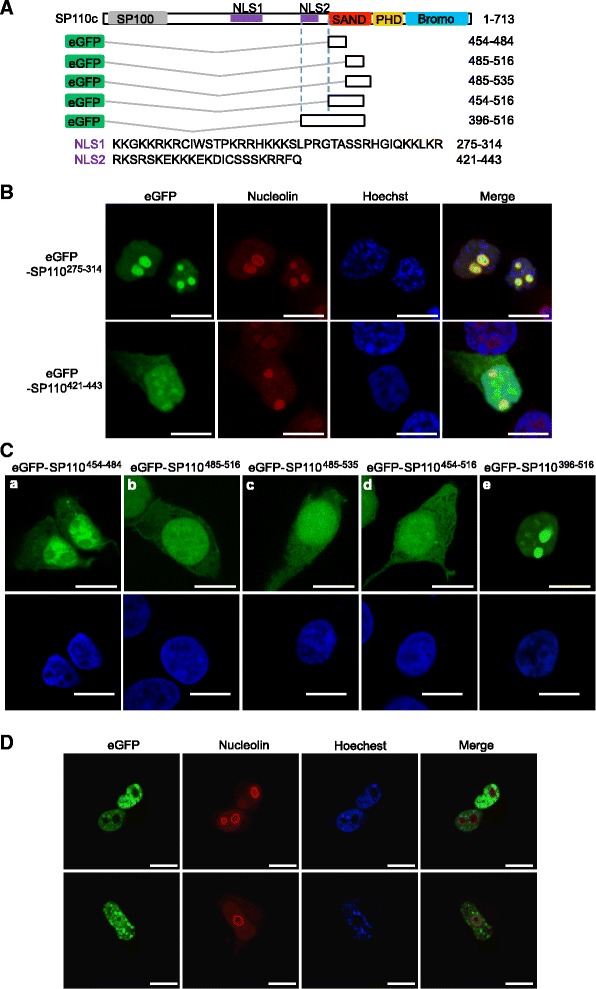
Identification of nuclear localization signals in SP110. **a** Structure of the SP110 protein, showing the positions of the nuclear localization signals (NLSs), and schematic representation of eGFP fusion proteins that contain fragments of the SAND domain of SP110. **b** and **c** HEK293T cells were transfected with the indicated constructs, and the disparate localization of eGFP fusion proteins containing NLSs (**b**) and the SAND fragments (**c**, upper panels) was visualized by confocal microscopy at 2 days post-transfection. Scale bars: 10 μm. **d** H1299 cells were transfected with the construct expressing the eGFP-SP110b fusion protein, and the nuclear and nucleolar localizations of the protein were visualized by confocal microscopy at 2 days post-transfection. The upper and lower panels are representative of the eGFP-SP110b fusion protein that is localized in the nucleoplasm and the nucleolus, respectively. Scale bars: 10 μm. The cells were probed with an anti-nucleolin antibody to identify nucleoli (**b** and **d**) and subjected to Hoechst staining to identify nuclei (**b**, **d**, and lower panels in **c**). All data represent at least 2 independent experiments

Prediction of the functional motifs in SP110 using the eukaryotic linear motif (ELM http://elm.eu.org) resource [32] showed several clusters of basic amino acids within the middle region of the protein and two potential NLSs (NLS1: 275–314, SP110^275–314^; NLS2: 421–443, SP110^421–443^), as delineated in Fig. 3a. NLS1, which is pretty much close to the putative NLS reported previously [28], directed the eGFP fusion protein to the nucleolus, while the fusion of eGFP to NLS2 predominantly resulted in nucleoplasmic, not nucleolar, localization (Fig. 3b). These observations indicate that NLS1, located between amino acids 275 and 314 of SP110, is a nucleolar localization signal (NoLS).

Since the SAND domain fused to eGFP was localized to the nucleus (Fig. 2d), we also analyzed the SAND domain using deletion mutations to identify the potential NLSs within this region. However, although the distribution of the deletion mutants is mainly in the nucleus, the proteins appear to be more frequently observed in cytosol than the eGFP fusion protein containing the full-length SAND domain (Fig. 3c, (a-d) and Fig. 2d). Interestingly, a fragment (amino acids 396–516) that contains NLS2 and a partial SAND domain directed the eGFP fusion protein GFP-SP110^396–516^ to the nucleus and nucleolus (Fig. 3c, (e)), suggesting that the NLS2 and SAND domain may form a more stable structure for nuclear and nucleolar targeting. In our experimental conditions, none of the SP110 isoforms were observed in the nucleolus when the proteins were over-expressed in HEK293T cells, even though an NoLS was present in SP110. However, we found that full-length SP110b was localized to the nucleolus at a low frequency (less than 5%) when the protein was expressed in H1299 cancer cells (Fig. 3d, lower panels).

### The SP110 NoLS contains one monopartite and one bipartite NLSs

**Fig. 4:**
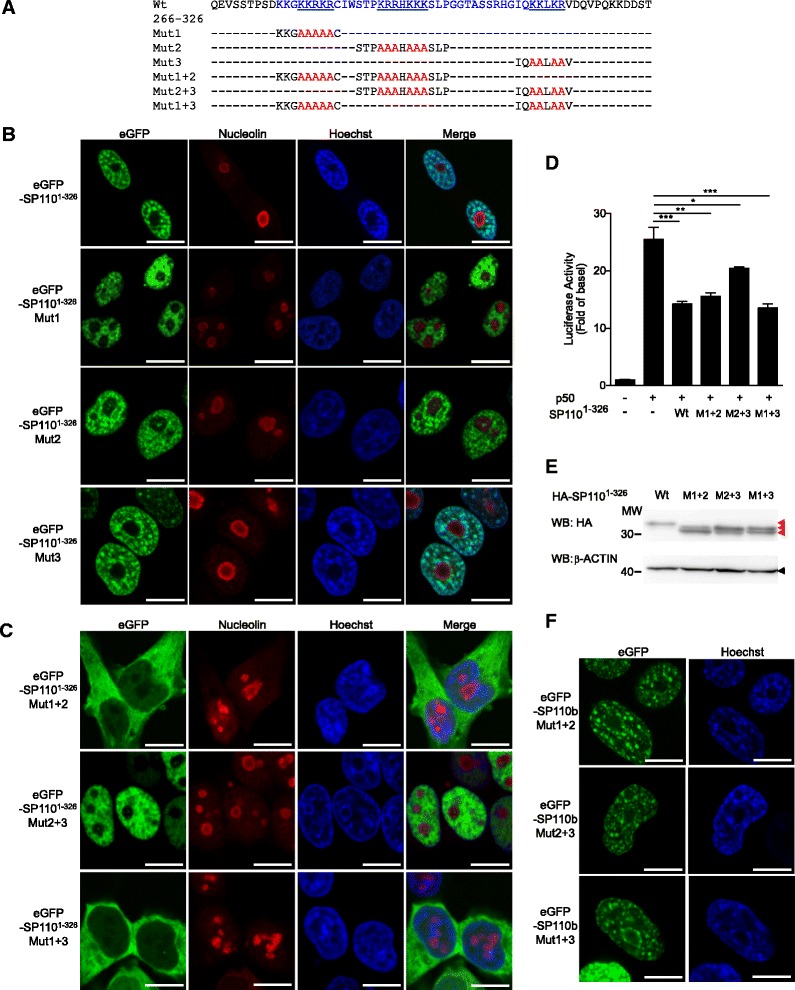
Identification of a nucleolar localization signal in SP110. **a** Amino acid sequence alignment of the wild-type and mutated nucleolar localization signals (NoLSs) of SP110. The sequence between amino acids 275 and 314 of SP110 identified as a potential NoLS is indicated in blue. Three lysine/arginine-rich (KR) clusters in the region are underlined. **b** and **c** HEK293T cells were transfected with the indicated constructs, and the localization of eGFP-SP110^1–326^ fusion proteins that contain the wild-type or mutated NoLS was visualized by confocal microscopy at 2 days post-transfection. The cells were probed with the anti-nucleolin antibody to identify nucleoli and subjected to Hoechst staining to identify nuclei. Scale bars: 10 μm. **d** HEK293T cells were co-transfected with pGL3-TNFα promoter-F.Luc, pSV40-R.Luc, and the indicated constructs, and the relative Luc values for the TNFα promoter were measured 2 days after co-transfection. The empty vector was included to the left as a negative control. The data are presented as the mean ± SD. Statistical significance of the difference between two sample groups was calculated using a two-tailed unpaired *t*-test. *P < 0.05; **P < 0.01; ***P < 0.001. **e** HEK293T cells were transfected with the respective constructs, and whole-cell HEK293T extracts were analyzed by Western blot (WB) with the indicated antibodies at 2 days post-transfection. Red and black triangles indicate HA-SP110^1–326^ and β-ACTIN, respectively. **f** HEK293T cells were transfected with the indicated constructs, and the cellular distribution of eGFP-SP110b fusion proteins that contain a mutated NoLS was visualized using confocal microscopy at 2 days post-transfection (left panels). The cells were also subjected to Hoechst staining to identify nuclei (right panels). Scale bars: 10 μm. In (**d** and **e**), M1 + 2, M2 + 3 and M1 + 3 indicate Mut1 + 2, Mut2 + 3 and Mut1 + 3, respectively. All data represent at least 2 independent experiments

The prospective NoLS (that is NLS1) in the region between amino acids 275 and 314 of SP110 contains three lysine/arginine-rich (KR) clusters, as shown in Fig. 4a. To more precisely characterize the roles of these KR clusters in the function of the NoLS, a set of eGFP-SP110^1–326^ fusion proteins that contain either the wild-type NoLS or a mutant NoLS was generated and expressed (Fig. 4a). eGFP-SP110^1–326^ proteins that contain the wild-type NoLS or a mutant NoLS in which a single KR cluster was mutated (Mut1, Mut2, or Mut3) were localized to the nucleus and exhibited similar nuclear localization patterns to the full-length SP110 proteins (Figs. 2b and 4b). The two eGFP-SP110^1–326^ proteins harboring NoLS mutations in two of the three KR clusters (Mut1 + 2 or Mut1 + 3 but not Mut2 + 3) were localized to the cytoplasm, as was eGFP-SP110^1–276^ (Fig. 4c). The results indicated that the potential NoLS in SP110 consists of one monopartite NLS (cluster 1) and one bipartite NLS (clusters 2 and 3) [33–35] and that eGFP-SP110^1–326^ is not able to enter the nucleus when both NLSs are disrupted (Mut1 + 2 or Mut1 + 3). Moreover, we demonstrated that the p50 dimer-driven TNFα promoter activity was suppressed by SP110^1–326^, as well as by proteins containing a NoLS with mutations in two KR clusters (Mut1 + 2, Mut2 + 3, or Mut1 + 3) (Fig. 4d), despite similar expression levels (Fig. 4e).

To further examine the effects of the NoLS mutations on the cellular distribution of SP110b, full-length eGFP-SP110b proteins containing NoLS mutations (Mut1 + 2, Mut2 + 3, and Mut1 + 3) were generated and expressed. The results showed that the NoLS mutations did not affect the pattern and localization of full-length eGFP-SP110b protein (Fig. 4f). This suggests that a region other than the NoLS, such as NLS2, can direct nuclear localization of full-length eGFP-SP110b protein containing NoLS mutations.

### The N-terminal region of SP110 interacts with p50

Although the expression of the N-terminal fragment (amino acids 1–276) of SP110 (SP110^1–276^) that does not contain any nuclear localization signals resulted in localization of eGFP-SP110^1–276^ to the cytoplasm (Fig. 2c), the protein fragment down-regulated p50 dimer-driven TNFα transcription in the nucleus (Fig. 1c). This result suggested that the N-terminal fragment of SP110 may mediate unknown mechanisms other than directly interacting with p50 complex in the nucleus, ultimately resulting in the down-regulation of p50 dimer-driven TNFα promoter activity.

**Fig. 5:**
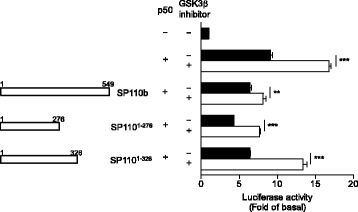
SP110-mediated down-regulation of NF-κB activity is independent of GSK3β. HEK293T cells were co-transfected with pGL3-TNFα promoter-F.Luc, pSV40-R.Luc, and the indicated constructs, and cultured in the absence or presence of GSK3β inhibitor. The relative Luc values for the TNFα promoter were then measured 2 days after co-transfection. The empty vector negative control is shown at the top. The data are presented as the mean ± SD. Statistical significance of the difference between two sample groups was calculated using a two-tailed unpaired *t*-test. **P < 0.01; ***P < 0.001. The data represent 3 independent experiments

To investigate the function of the N-terminal region of SP110, we analyzed this region using the ELM database (http://elm.eu.org/) and found that potential phosphorylation sites that may also affect its interactions with other proteins are clustered within the N-terminal portion of SP110. Among these phosphorylation sites, several consensus sequences for glycogen synthase kinase 3β (GSK3β) substrate (Ser/Thr-X-X-X-Ser/Thr, where X is any residue) [36] were predicted in the region (Table 1). We thus speculated that SP110 proteins may be phosphorylated by GSK3β, a serine/threonine kinase with a diverse range of cellular targets, including transcription factors, components of the cell cycle, and proteins involved in microtubule dynamics [37, 38], and that phosphorylation at these sites may affect SP110 functions. Using a small molecule GSK3β inhibitor, we determined whether GSK3β is involved in SP110-mediated down-regulation of TNFα promoter activity. The results demonstrated that GSK3β negatively regulates p50 dimer-driven TNFα promoter activity and that in the presence of SP110 proteins, GSK3β inhibition does not restore TNFα transcription to the levels driven by p50 in the absence of SP110 proteins (Fig. 5). Our findings indicate that GSK3β has only a partial, if any*,* effect on SP110 (full-length SP110b and deletion mutants)-mediated down-regulation of p50 dimer-driven TNFα promoter activity.

**Table 1:** Sequence motifs in SP110 predicted for the substrate recognition sites of GSK3β

Matched Sequence	Positions
AEGSSLHT	128–135
PRVSEPGT	154–161
PGTSSQQS	159–166
EILSESPS	168–175
LSESPSPS	170–177
EGRSTSVT	189–196
EMPSLLTS	212–219
MPHSPLGS	241–248
QEVSSTPS	266–273
KDDSTCNS	322–329
DDSTCNST	323–330
TCNSTVET	326–333
KDDSTWNS	399–406

**Fig. 6:**
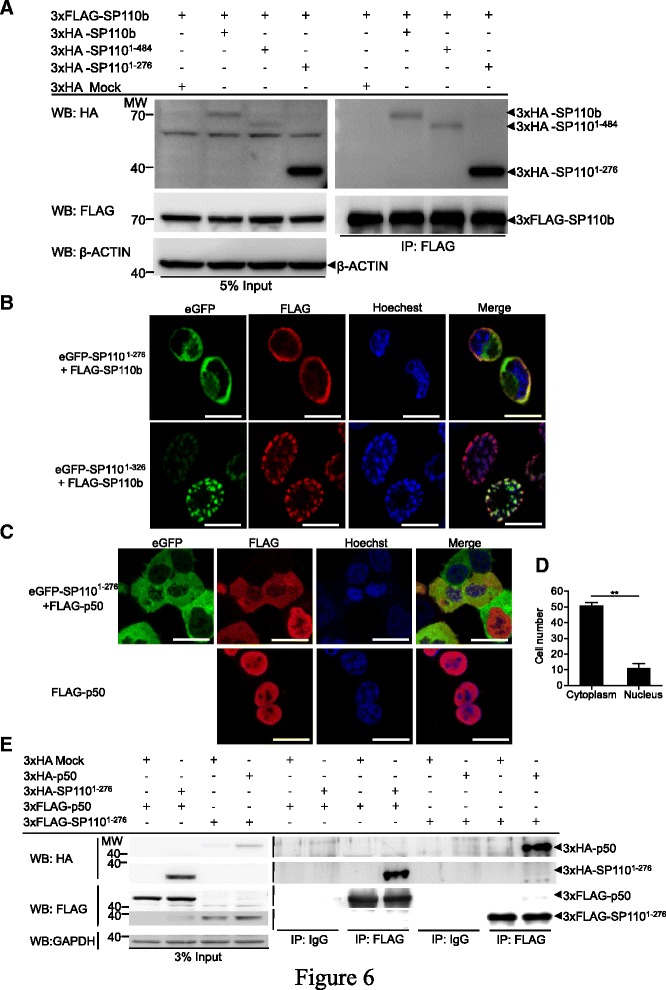
Interaction of the N-terminal region of SP110 with SP110b and NF-κB proteins. **a** HEK293T cells were co-transfected with the respective constructs, and whole-cell HEK293T extracts were subjected to immunoprecipitation (IP) using an anti-FLAG antibody and analyzed by WB with the indicated antibodies at 2 days post-transfection. **b** and **c** HEK293T cells were co-transfected with the indicated constructs, and the cellular distribution of eGFP-SP110 fusion proteins that contain deletion mutants of SP110 protein (**b** and **c**), FLAG-SP110b (**b**) and FLAG-p50 (**c**) was visualized using confocal microscopy at 2 days post-transfection. Scale bars: 10 μm. The cells were probed with an anti-FLAG antibody to identify FLAG-SP110b (**b**) and FLAG-p50 (**c**), respectively, and subjected to Hoechst staining to identify nuclei. **d** HEK293T cells were co-transfected with the constructs expressing eGFP-SP110^1–276^ and FLAG-p50, and the cells were probed with an anti-FLAG antibody to identify FLAG-p50 and visualized using microscopy at 2 days post-transfection. The numbers of cells with p50 cytoplasmic or nuclear distribution were determined by counting at least 60 cells per field from two randomly selected microscopic fields. Mean number of cells with p50 in cytoplasm or nucleus was shown. The data are presented as the mean ± SD. Statistical significance of the difference between two sample groups was calculated using a two-tailed unpaired *t*-test. **P < 0.01. **e** HEK293T cells were co-transfected with the respective constructs, and whole-cell HEK293T extracts were subjected to IP using an anti-FLAG or control IgG antibody and analyzed by WB with the indicated antibodies at 2 days post-transfection. All data represent at least 2 independent experiments

We next demonstrated that the N-terminal region (amino acids 1–276) self-interacted with SP110b (Fig. 6a) and that this interaction resulted in cytoplasmic sequestration of SP110b (Fig. 6b). Furthermore, SP110^1–276^-mediated cytoplasmic sequestration was also observed for p50, which is mainly localized to the nucleus (Fig. 6c and d). The interaction between SP110^1–276^ and p50 was also demonstrated by co-immunoprecipitation (co-IP) and further confirmed by reciprocal co-IP (Fig. 6e). These results indicate that the interaction of the N-terminal fragment (amino acids 1–276) of SP110 (SP110^1–276^) with p50 sequesters p50 in the cytoplasm, resulting in the down-regulation of the p50 dimer-driven TNFα transcription in the nucleus. These findings also suggest that the N-terminal region of SP110 (1–276) can sequester proteins that interact with it in specific subcellular compartments where the full-length SP110 located.

## Discussion

NF-κB proteins are capable of inducing hundreds of genes that have tremendously diverse and often opposing biological functions. These sequence-specific transcription factors, via cooperating with other transcription factors and co-regulators, induce specific transcriptional activity at each promoter following binding to κB DNA response elements [39–43]. Given that NF-κB cooperates with many other transcriptional regulators, NF-κB-mediated transcription can be determined by a characteristic NF-κB containing complex. To investigate whether SP110 protein regulates transcriptional activity of NF-κB, two luciferase reporter assay systems were used in this study. In one system, the luciferase reporter gene is driven by a basic promoter element (TATA box) linked to 5 NF-κB binding element repeats (κB sites); in the other one, the luciferase gene is driven by a segment of the *TNFα* promoter sequence that contains seven potential κB-binding sites [16]. The reporter plasmid was cotransfected in HEK293T cells with different combinations of expression vectors of NF-κB subunits p65 or p50. The NF-κB p65/p50 and p50/p50 are two of the most abundant NF-κB dimers that are present in cells [44]. In vitro studies have demonstrated that κB-site-dependent transcriptional activation can be induced by p50 dimers [45, 46], probably by cooperating with other transcriptional co-regulators [47, 48]. Therefore, the luciferase activity in the cells was induced by overexpression of NF-κB dimers p65/p50 or p50/p50 without priming the cells. The data obtained from luciferase reporter assays demonstrated that the three SP110 isoforms affected NF-κB activity differently (Fig. 1a and b), suggesting that the SP110 isoforms may have distinct cellular functions. This evidence is similar to previous findings demonstrating that SP110b is a potent transcriptional co-repressor of the retinoic acid receptor alpha (RARα), while SP110a functions as a co-activator [22]. We demonstrated that the N-terminal fragment (amino acids 1–276) of SP110 (SP110^1–276^) is sufficient to down-regulate p50 dimer-driven TNFα promoter activity. However, this suppressive effect of the N-terminal region may be influenced by the various C-terminal regions of each SP110 isoform. In the C-terminal regions of SP110a and SP110c, the PHD finger, which interacts with methylated or unmodified lysines of histone tails, has been shown to be involved in chromatin-mediated transcriptional regulation [49, 50], and the BRD module, which recognizes acetyl-lysine residues of targets, is also involved in regulating gene transcription [51, 52]. Therefore, the existence of a PHD/BRD tandem module in the C-terminal regions of SP110a and SP110c that is also present in several chromatin-associated proteins, such as transcription intermediary factor 1α (TIF1α) [53], tripartite motif (TRIM)-containing protein 33 (TRIM33) [54], and human transcriptional co-activator CBP [55], is likely to contribute to the transcription-regulating properties of SP110 isoforms.

Protein structural analyses demonstrated that SP110b contains an SP100-like nuclear body protein interaction domain, followed by an LXXLL nuclear receptor binding motif and a chromatin-associating SAND domain. It has been reported that PIAS3, a protein inhibitor of activated STAT3, suppresses NF-κB-mediated transcription by interacting with p65 via its LXXLL-motif [56]. Given that SP110 proteins also contain an LXXLL-motif (amino acids 525 to 529; LGELL), it raised the possibility that SP110 may interact with p65 via this motif thereby regulating NF-κB-mediated transcription. However, our data determined that the region between amino acids 1 and 276 (located outside the LXXLL-motif in all SP110 isoforms) was sufficient to suppress NF-κB-mediated TNFα induction (Fig. 1c), suggesting that SP110 proteins use an unknown mechanism independent of the LXXLL motif to regulate NF-κB activity. Given that the N-terminal SP100 domain of human SP100 is required for its dimerization [57], as well as its ability to interact with other cellular proteins, such as ETS1, HP1 (heterochromatin protein 1), and HIPK2 (homeodomain-interacting protein kinase-2) [58–60], we investigated whether SP110^1–276^ that contains an SP100 domain could interact with NF-κB. Our results demonstrated that the SP100 domain not only self-interacted with SP110b but also interacted with and sequestered p50 protein in the cytoplasm (Fig. 6). This result suggests that SP110, via the region between amino acids 1 and 276, interacts with p50 and sequesters it in particular subcellular compartments, thereby interrupting the formation of a complex consisting of p50 and other components in the nucleus. This interaction contributes to the SP110b-mediated down-regulation of the p50 dimer-driven TNFα transcription.

Using eGFP fusion proteins that contain a series of deletion mutants of SP110, we showed that amino acids 275–314 of SP110 are crucial for the targeting of eGFP-SP110 fusion protein to the nucleolus. This sequence includes three KR-rich clusters, which constitute one consensus monopartite NLS (cluster 1) and one bipartite NLS (clusters 2 and 3) [33–35]. The results from the analysis of the cluster mutants showed that each NLS is sufficient to target a protein to the nucleus (Fig. 4c). When the identified motif was fused to eGFP, it was predominantly localized to the nucleolus. However, only a small percentage of full-length SP110 containing the NoLS was observed in the nucleolus of H1299 cells in our experiment. One possible explanation for this result is that although SP110 contains an NoLS, it is normally sequestered away from the nucleolus and localized to the nucleoplasm as a result of interacting with other nuclear factors through regions outside of this motif. In the nucleoplasm, SP110 is able to form nuclear speckles and participate in transcription and pre-mRNA processing. However, the motif located within amino acids 275–314 of SP110 may target the protein to the nucleolus probably as cells encounter various specific physiological and stressful conditions. Given that the nucleolus is plurifunctional and involved in many cellular processes other than ribosome biogenesis [61], nucleolar localization of SP110 may facilitate the execution of specific functions by SP110 and the regulation of certain cellular processes by this protein. Previous findings have likewise shown that human MDM2 containing a C-terminal NoLS is normally localized to the nucleoplasm but is redirected to the nucleolus via interaction with ARF (p14^ARF^) in response to stress signals, thereby preventing MDM2-triggered degradation of p53 and leading to p53 activation in the nucleoplasm [62, 63]. It remains to be determined how the nucleolar localization of SP110 is regulated.

## Conclusions

The present study identifies functional domains within SP110 required for its transcriptional regulatory function and cellular translocation. The results indicate that the N-terminal region (amino acids 1–276) of SP110 plays a key role in SP110-mediated down-regulation of the TNFα promoter activity, suggesting that this region may serve as a template for the design and development of peptide-based therapeutics. In addition, the identification of nuclear and nucleolar localization signals in SP110 suggests that SP110 performs additional and currently unknown functions when localized to different subcellular compartments. It will be of utmost importance to determine whether proper compartmentalization of SP110 is relevant to the SP110 functions as well as how it is regulated. The findings in this study highlight that the multidomain structure of SP110 may be essential to its versatile functions, indicating that more in-depth research into the biochemical and biophysical properties of this protein is warranted.

## Additional files

Additional file 1: **Table S1.** A list of primers used for plasmid construction. **Table S2.** A list of primers used for generating deletion mutants of SP110. **Table S3.** A list of primers used for site-directed mutagenesis of SP110. **Table S4.** A list of antibodies used in this study. (PDF 45 kb)

Additional file 2: **Figure S1.** Subcellular localization of FLAG-tagged wild-type SP110 or SP110 mutant proteins. (a) Schematic representation of FLAG-tagged proteins containing SP110 deletion mutants. SP100: SP100 domain; SAND: SAND domain; PHD: PHD finger; Bromo: Bromodomain. (b-c) HEK293T cells were transfected with the indicated constructs, and the cellular distribution of FLAG-tagged SP110 proteins (wild-type (b) and mutated forms (c)) was visualized using confocal microscopy at 2 days post-transfection (upper panels). The cells were also subjected to Hoechst staining to identify nuclei (lower panels). Scale bars: 10 μm. The data represent 3 independent experiments. (PDF 738 kb)

## Abbreviations

BRD: Bromodomain
Dox: Doxycycline
eGFP: Enhanced green fluorescent protein
ELM: Eukaryotic linear motif
F.Luc: Firefly luciferase
GSK3β: Glycogen synthase kinase 3β
IFN: Interferon
IP: Immunoprecipitation
*Ipr1*: Intracellular pathogen resistance 1
*Mtb*: *Mycobacterium tuberculosis*
NB: Nuclear body
NF-κB: Nuclear factor-κB
NLS: Nuclear localization signal
NoLS: Nucleolar localization signal
PCR: Polymerase chain reaction
PHD: Plant homeodomain
R.Luc: *Renilla* luciferase
*sst1*: Supersusceptibility to tuberculosis 1
TB: Tuberculosis
TNFα: Tumor necrosis factor-α
WB: Western blot
