# Supplementary material for: Functional domains of SP110 that modulate its transcriptional regulatory function and cellular translocation
Source: J Biomed Sci. 2018 Apr 11;25:34. doi: 10.1186/s12929-018-0434-4 (PMC5894228; doi:10.1186/s12929-018-0434-4)
Supplement: Supplementary file 1 — Table S1. A list of primers used for plasmid construction. Table S2. A list of primers used for generating deletion mutants of SP110. Table S3. A list of primers used for site-directed mutagenesis of SP110. Table S4. A list of antibodies used in this study. (PDF 45 kb) [file 12929_2018_434_MOESM1_ESM.pdf]

Table S1 A list of primers used for plasmid construction

Primers	Sequence	Note
SP110-XhoI-F	5'-TTTTTTctcgag**ATG**TTCACCATGACAAGAGC-3'	Construction of SP110b in pHAGE vector
SP110b-BglII-SalI-R	5'-ATATTagatctgtcgac**TCA**CTTGCTATTTAACTCTCTC-3'	
SP110-HindIII-F	5'-TTTTTaagctt**ATG**TTCACCATGACAAGAGC-3'	Construction of SP110a/b/c in pcDNA-3xFLAG and pcDNA-3xHA vectors
SP110a-NotI-R	5'-ATATTgcggccgc**TCA**AGGAAGAGTCCAGAAAC-3'	
SP110b-NotI-R	5'-TTATTgcggccgc**TCA**CTTGCTATTTAACTCTCTC-3'	
RELA-KpnI-F	5'-AATAggtaccACC**ATG**GACGAACTGTTC-3'	Construction of p65 or p50 in pcDNA-3xFLAG and pcDNA-3xHA vectors
RELA-XbaI-R	5'-AATAtctaga**TTA**GGAGCTGATCTGACTCA-3'	
p50-KpnI-F	5'-AAggtacc**ATG**GCAGAAGATGATCCATATTTGGG-3'	
p50-XhoI-R	5'-AATTctcgag**CTA**TTTCCCAAAGAGGTTTACAGTG-3'	
TNF-1786-NheI-F	5'-TCAGGGgctagcACCACCTCTCCTTTGGC-3'	Construction of TNF promoter in pGL3-basic vector
TNF+175-HindIII-R	5'-CATGCTTTCAGTaagcttGGTGTCCTTTCCAG-3'	

Table S2 A list of primers used for generating deletion mutants of SP110

Primers	Sequence
SP110-XhoI-F	5'-TTTTTT**CTCGAG**ATGTTCACCATGACAAGAGC-3'
SP110^{276}-BamHI-SalI-TAG-R	5'-TTTTTT**GGATCCGTCGAC**CTATTTCTTGTCTGAAGGTGTG-3'
SP110^{256}-XhoI-F	5'-TTTTTT**CTCGAG**TCTCCAGAACCAAATGACC-3'
SP110^{484}-BglII-SalI-TGA-BamHI-R	5'-TTTTTT**AGATCTGTCGAC**TCA**GGATCC**GTGTTTCATTTC-3'
SP110^{454}-XhoI-F	5'-TTTTTT**CTCGAG**AGTGACACTGTGGATTTTC-3'
SP110^{689}-BglII-SalI-R	5'-ATATT**AGATCTGTCGAC**TCAAGGAAGAGTCCAGAAAC-3'
SP110^{549}-BglII-SalI-R	5'-ATATT**AGATCTGTCGAC**TCACTTGCTATTTAACTCTCTC-3'
SP110^{713}-BglII-SalI-R	5'-TTTTTT**AGATCTGTCGAC**TCAAGGAAGAGTCCAGAA-3'
SP110^{535}-BglII-SalI-TGA-R	5'-TTTTTT**AGATCTGTCGAC**TCAATCCGAGTTTTTCCGCTT-3'
SP110^{517}-Xho-F	5'-TTTTTT**CTCGAG**AATATACGTTGTGAAGGAAT-3'
SP110^{589}-XhoI-F	5'-AAAAAA**CTCGAG**CATCATGTATCTAAGACCC-3'
MyD88-111-KpnI-F	5'-AATA**GGTACC**GACGACGTGCTGCTGGAGC-3'
MyD88-121-XhoI-R	5'-AATA**CTCGAG**TCAAATGCTGGGTCCCAGCTCCA-3'

Table S3 A list of primers used for site-directed mutagenesis of SP110

Primers	Sequence
Mut1 (1-326)-Sense	5'-CAGACAAGAAAGGA**GCTGCCGCAGCGGCT**TGTATCTGGTCAAC-3'
Mut1(1-326)-Anti-Sense	5'-GTTGACCAGATACA**AGCCGCTGCGGCAGC**TCCTTTCTTGTCTG-3'
Mut2 (1-326)-Sense	5'-CTGGTCAACTCCA**GCTGCCGCA**CAT**GCTGCCGCA**AGCCTCCCAGGAGG-3'
Mut2 (1-326)-Anti-Sense	5'-CCTCCTGGGAGGCT**TGCGGCAGC**ATG**TGCGGCAGC**TGGAGTTGACCAG-3'
Mut3 (1-326)-Sense	5'-GACACGGAATCCAA**GCTGCC**CTC**GCTGCC**GTGGATCAGGTTCC-3'
Mut3 (1-326)-Anti-Sense	5'-GGAACCTGATCCAC**GGCAGC**GAG**GGCAGC**TTGGATTCCGTGTC-3'

Table S4 A list of antibodies used in this study

Antibodies	Company	Catalog number
HA	GeneTex	#GTX29110
β-Actin	Millipore	#MAB1501
FLAG M2	Sigma-Aldrich	#F3165 & F7425
Nucleolin	Invitrogen	#39-6400
Glyceraldehyde-3-phosphate dehydrogenase (GAPDH)	GeneTex	#GTX100118
