# Supplementary figures and images for: Functional domains of SP110 that modulate its transcriptional regulatory function and cellular translocation
Source: J Biomed Sci. 2018 Apr 11;25:34. doi: 10.1186/s12929-018-0434-4 (PMC5894228; doi:10.1186/s12929-018-0434-4)

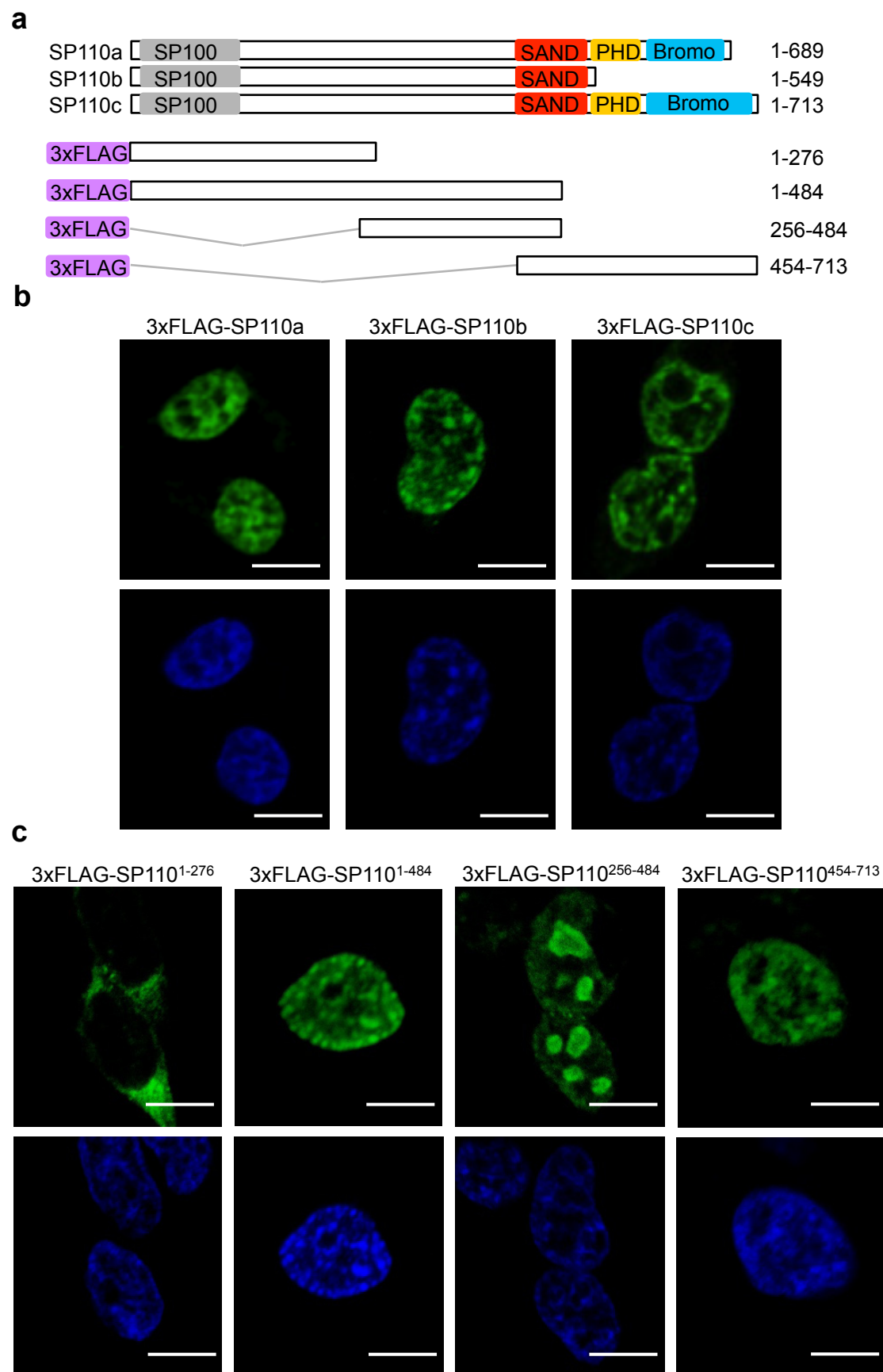

Figure S1

Supplement: Supplementary file 2 — Figure S1. Subcellular localization of FLAG-tagged wild-type SP110 or SP110 mutant proteins. (a) Schematic representation of FLAG-tagged proteins containing SP110 deletion mutants. SP100: SP100 domain; SAND: SAND domain; PHD: PHD finger; Bromo: Bromodomain. (b-c) HEK293T cells were transfected with the indicated constructs, and the cellular distribution of FLAG-tagged SP110 proteins (wild-type (b) and mutated forms (c)) was visualized using confocal microscopy at 2 days post-transfection (upper panels). The cells were also subjected to Hoechst staining to identify nuclei (lower panels). Scale bars: 10 μm. The data represent 3 independent experiments. (PDF 738 kb) [file 12929_2018_434_MOESM2_ESM.pdf]
